# Off-label drugs use in pediatric palliative care

**DOI:** 10.1186/s13052-018-0584-8

**Published:** 2018-11-29

**Authors:** Lucia De Zen, Federico Marchetti, Egidio Barbi, Franca Benini

**Affiliations:** 1 Pediatric Home Assistance and Palliative Care, Department of Pediatrics, AAS5 Friuli Occidentale, via Montereale 24, 33170 Pordenone, Italy; 2 Department of Pediatrics, S. Maria delle Croci Hospital, AUSL della Romagna, Ravenna, Italy; 3 Institute for Maternal and Child Health - IRCCS “Burlo Garofolo”, Trieste, Italy; 4 University of Trieste, Trieste, Italy; 5 Paediatric Palliative Care - Pain Service, Department of Women’s Children’s Health, University of Padua, Padova, Italy

**Keywords:** Pediatric palliative care, Pain, Off-label drugs

## Abstract

**Background:**

Paediatric palliative care (PPC) aim to ensure the control of symptoms and the best possible quality of life for patients whose underlying disease, characterized by an unstoppable evolution and negative prognosis, no longer responds to specific treatments. The scientific evidence in this context are very deficient and, in order to obtain welfare objectives consistent with the situation, in the overwhelming majority of cases the prescription of drugs is off-label for indication of use and/or for age and/or for way of administration and/or formulation.

The Agenzia Italiana del Farmaco - AIFA and the Italian Society of Palliative Care (Società Italiana di Cure Palliative - SICP), under a dedicated working group, wrote a document that collects the scientific evidence available to support the off-label use of medicines more frequently used in PPC. The goal is to certify the consolidated off-label use of these drugs and propose their use under the Law 648/96, in the absence of data from its pivotal clinical trials.

Aim of the commentary is to report the conditions for this important work and to present the 10 drugs, usually used off-label in PPC and in pain therapy, now included in Law 648/96.

**Conclusion:**

This work is deemed essential to resolve, at least in part, the lack of availability of medicines researched and approved.

## Introduction

### Off-label use of drugs in pediatrics

On January 26th 2007, the European Regulation on medicinal products for paediatric use (Regulation EC No 1901/2006) [1] entered into force in all the European Union countries. The aims of this regulation are to facilitate the development and the accessibility to medications specifically designed for children from 0 to 18 years old, to ensure that medicinal products used in paediatric population are subject to high quality ethical research and appropriately authorized for use in children, as well as to increase the informations availability on use of medicines for children [2].

However despite the European Regulation and initiatives put forward in this area by Agenzia Italiana del Farmaco (AIFA), still only one third of drugs available to the adult range gets to the pediatric patient and sometimes only after many years. The analysis of the current situation shows that many new drugs and most molecules already available since long time on the market, are not registered for pediatric use.

It follows that, in clinical practice, children are often treated with medications designed and tested only in the adults, according to procedures and indications not specifically envisaged nor registered for children (off-label use of the drug). Using a drug off-label means prescribing it in conditions that differ from those for which it has been authorized, in terms of age, dosage regimen (dose or frequency of administration), therapeutic indication, route of administration and formulation.

The off-label and unlicensed classification methods varied, making results difficult to compare [3–5]. Many studies reported high rates of off-label (9 to 78.7%) and unlicensed (0.3 to 35%) drug use in different pediatric patient settings [5]. The extent of paediatric unlicensed/off label use is higher in neonatal and paediatric intensive care units and oncology wards, compared with primary care [4]. On the paediatric hospital wards, off-label/unlicensed prescriptions ranged from 16 to 62% [3]. In the neonatal wards, rates ranged from 55 to 80% [3]. In the community setting, rates ranged from 11 to 37% [3].

This lack of adequate registration is caused by different issues:

- There are problems of numerosity and heterogeneity of patients and clinical situations: between 0 and 18 years there are at least three different sub-populations (infants, children and adolescents) with biological and metabolic characteristics significantly different from each other, and therefore requiring specific studies and experiments [6].
- There are ethical issues: there is in fact a kind of ethical bias to expose children to experimental clinics, although, on the contrary, this may go to harm their own interests, since it precludes the possibility of developing drugs tailored to their specific needs [7].
- .There are finally economic reasons determined by the costs of the trials and the limited economic return: a situation that discourages investment by the pharmaceutical industry.

Since publication of the 2002 statement from the American Academy of Pediatrics on the off-label use of drugs [8], the number of drugs approved with pediatric indications or expanded labeling that informs drug use in pediatric patients has substantially increased [9–11]. However, despite this success and advances in both basic science and clinical trials in pediatrics, off-label drug use remains a common and important issue for children and adolescents [12].

Off-label use is in many situations the only therapeutic option [12–14]. Such use has in clinical practice a “crucial” and, in many situations, not deferrable role and, although unauthorized, has frequently long periods of pediatric use (clinical practice statement), as well as studies and reports in the literature that strongly support drug efficiency and safety [12, 14].

Remarkably off-label prescription has clinical, legal and ethical implications to consider [12] the chance of making mistakes in the definition of a treatment is greater, it involves the direct assumption of responsibility by the prescriber (both in terms of efficacy and possible adverse effects), it requires informed consent by a person conducting the parental authority and has an impact on drug reimbursement.

The issue of the off-label use of drugs in Pediatrics is further amplified in certain areas, where even more problems related to the difficulty of developing clinical trials for specific type of patients, features of situations and/or newness of the problema may arise [12, 15]. For example, these critical issues are greater in neonatology, in rare and complex diseases, in children with life threatening/limiting disease and/or in terminal illness, where trials are rarely conducted both for the low numerosity and high heterogeneity of the population, both for ethical reasons and sometimes for lack of economic resources.

The result is that the off-label use of drugs in these conditions represents the standard of treatment, consolidated and time honoured in the management of the young patient. In these areas the treatment choices are made on the basis of an established practice, often deduced from experience/studies completed in adults, and limited pediatric specific evidence. This is the case of pediatric palliative care [16].

### Pediatric palliative care

In recent years, a slow but continuous change of the care needs of the ill infant/child/adolescent took place: new types of patients, new situations and new goals of “health”. Certainly one of these new fields is represented by the needs of Palliative care in the pediatric population.

Pediatric Palliative Care (PPC) are that part of pediatric medicine that takes care of children with incurable disease and/or severe disabilities: the goal of palliative care is the quality of young patient’s life and symptom control [17]. Home is, in the vast majority of cases, the chosen and ideal place for care and treatment. The spectrum of diseases potentially eligible at PPC is heterogeneous and broad (neurological diseases, oncology, cardiology, respiratory, muscular, metabolic, infectious, developmental disorders, chromosomal, post anoxic) and heterogeneous and large is also the spectrum of needs, clinical and not, that they trigger, and the modality needed for the care of the patient. It is estimated that in Italy children eligible to PPC are about 30,000 [18].

The main goal for all these children is alleviating suffering and pain, while addressing and containing all the other stressful symptoms and conditions. In these contexts the scientific evidence are scant and, in order to obtain welfare objectives consistent with the situation, the prescription of drugs is off-label in the overwhelming majority of cases, for indication use and/or for age and/or modality of administration and/or formulation [19]. Minors eligible at PPC are patients at high-complexity of assistance, affected by multiple pathologies which trigger complex symptoms that require polytherapy, frequently for long periods of time.

These are often patients with complex conditions which may limit the possibility of assuming drugs through routine routes of administration, requiring alternative ways such as the intranasal, the submucosal, the inhalatory one. Often these children may have cognitive and/or relational deficits which limit the possibility for sharing, actively participate or accept the management of a treatment plan.

In the majority of cases, the management of these infants/children/teenagers takes place at home and thus the medication management is settled into a different contest from that of a hospital or outpatient setting, with different standards of recording and monitoring.

### Off-label drugs and law 648/96

In Italy, the off-label use is governed by Law 648/96 that has identified a list of medications with a therapeutic indication other than that authorised, used in clinical practice for consolidated use and data from scientific literature. These drugs, once inserted in the list of medicinal products established by Law 648/96, are administered under the physician’s direct responsibility and can be reimbursed by the National Health Service [20].

In this list, there are drugs partially covering the needs of different areas of pediatric medicine such as drug listed for oncological, cardiovascular [21], gastrointestinal, anesthetics [22], anti-infective, dermatological, genitourinary tract medications and hormones.

This list lacks of some medicines frequently used to control pain and other relevant symptoms in the PPC.

Given the peculiar situations and cure aims that PPC promise, we identified a list of medications used in the PPC for which inclusion in the list established by Law 648/96, represents for patients, operators and all the health system a goal of efficacy, safety and equity.

For using such drugs, it remains mandatory to collect, by the legal representatives of the child, the informed consent at use. It’s also important to inform the child, with tool and modality appropriate to age, clinical situation and capacity of discernment, on medication and strategies of administration, in the perspective of sharing and participating to the treatment program.

## Aims

A list of drugs used off-label in pediatric palliative care (PPC) and in pain therapy, deemed essential to solve, at least in part, the low availability of medicinal products studied and approved in pediatric age, has been filled out. Ten drugs have been identified that routinely, for specific indications in PPC clinical practice, are used off-label with modality that are different from those for which they have been authorized in terms of age, dosage, therapeutic indication, route of administration and formulation.

In order to derive a proposal for inclusion in the list of medications dispensed in accordance with law 648/96, the work includes information about the scientific evidence to support the off-label use (literature, RCT, any clinical studies in course) and the use of the active ingredient in the off-label indication in other Member States (United Kingdom, with reference to the British National Formulary, BNF for children, Edition 2016–17).

The list has the aim to indicate the active ingredients, for each ATC class, which can be used in children under the CPP, although their use is not approved in childhood. For some of them, there is a rational use as supported by available evidence, even in the absence of controlled trials, frequently in case of old molecules or for objective difficulties in conducting pediatric clinical trial.

Some indications applied for inclusion in the list according to Law 648/96, refer to therapies that are used for short periods of time, in the course of exacerbations and/or end-of-life. Other requests instead, refer to prolonged use (months, years), during the course of taking charge in palliative care of the child with an incurable disease.

For each of the 10 identified active ingredients a single card was formalized that lists the specific indication of on-label demand in clinical practice, the rationale of the request, the evidence in support of the request, the current situation that is approved for use from leaflet, any additional notes [23]. The drugs detected are listed in Table 1.

**Table 1:** The 10 drugs singled out for an authorised “off-label” use in PPC

Drug	On-label use	Authorized l. 648/96 off-label use	Advised dosage
HYOSCINA BUTYLBROMIDE	Pill in child > 14 years, supp in child > 6 years: spastic– painful events of urinary and genital tract.	1. Iv administration for intestinal obstruction due to peritonitis in pediatric patients with cancer. 2. Iv administration for reduction of secretions and rattle in terminally ill patient.	Iv: Child 1 month-4 years: 300–500 micrograms/Kg 3–4 times a day (max. Per dose 5 mg) Child 5–11 years: 5–10 mg 3–4 times a day Child 12–17 years:10–20 mg 3–4 times a day [24]
DEXMEDETOMIDINE	Procedural analgo-sedation outside the operating room (Not Operating Room Anesthesia - NORA) in children with difficult airway management and child with seizure disorders who must undergo diagnostic studies for locating epileptogenic foci Analgo-sedation of critical infant and child in ICU, mechanically ventilated and poorly responsive to conventional analgo-sedation treatment.	1. Control of stressful symptoms from disease or procedure and fix sleep outside the ICU in patients in palliative care, not responsive to conventional therapies. 2. Intranasal route of administration.	Iv: 1 mcg/kg in a 10-min bolus, increased up to a maximum of 3 mcg/kg, and followed by a 1 mcg/kg/h infusion [25] In: 1 to 4 mcg/kg, eventually re-administered at 1 mcg/Kg [25]
FENTANYL	Premedication for any type of anesthesia (also local) both in the postoperative period as during surgery.	1. Transdermal, iv use for acute and/or chronic pain management from cancer and not, in children in PPC. 2. Transmucosal use for procedural/acute/breakthrough pain in PPC.	Transdermal: based on oral morphine dose equivalent (given at 24-h totals). Product monograph: oral morphine 45 mg = 12 mcg/h patch oral morphine < 90 mg = 25 mcg/h patch oral morphine 135–189 mg = 50 mcg/h patch oral morphine 225–314 mg = 75 mcg/h patch [26] Iv: Child > 6 months and < 50 Kg: bolus 0,5–1 mcg/Kg every 1–2 h, dose to be administered over at least 30 s; infusion 0,5–2 mcg/Kg/h Child > 50 Kg: bolus 25–50 mcg every 1–2 h, dose to be administered over at least 30 s; infusion 25–200 mcg/h [27] Transmucosal: Child 2–18 years and weiting > 10 Kg: 15–20 mcg/Kg as a single dose, titrated to a maximum dose of 400 mcg [26]
GABAPENTIN	Pill in child > 6 years: adjunctive therapy in the treatment of partial seizures in the presence or absence of secondary generalization. > 12 years: monotherapy in the treatment of partial seizures in the presence or absence of secondary generalization	Neuropathic or mixed pain in children older than 2 years in palliative care.	By mouth: Child > 2 years: Day 1: 10 mg/Kg (maximum single dose 300 mg) Day 2: 10 mg/Kg twice daily Day 3 onwards: 10 mg/Kg 3 times daily Increase further if necessary to a maximum of 20 mg/Kg/dose (maximum single dose 600 g) Child > 12 years: The maximum daily dose can be increased according to responde, up to e a maximum of 3600 mg/day [26]
KETAMINE	Im, iv and continuous infusion administering Use for induction and maintenance of general anesthesia from neonatal and premedication in children older than1 month.	1.Use in patients in PPC for managing procedural or mixed/neuropathic pain that does not respond to other therapy, alone or in combination/replacement for opioid analgesics. 2. Intranasal administration	Im: Neonate: 4 mg/Kg, adjusted according to response, a dose of 4 mg/Kg usually produces 15 min of surgical anaesthesia Child: 4–13 mg/Kg, adjusted according to response, a dose of 4 mg/Kg sufficient for some diagnostic procedures, a dose of 10 mg/Kg usually produces 12–25 min of surgical anaesthesia [24] Iv for short procedures: Neonate: 1–2 mg/Kg, adjusted according to response, to be given over at least 60 s, a dose of 1–2 mg/Kg produces 5-1o minutes of surgical anaesthesia Child 1 month-11 years: 1–2 mg/Kg, adjusted according to response, to be given over at least 60 s, a dose of 1–2 mg/Kg produces 5-1o minutes of surgical Child 12–17 years: 1–4,5 mg/Kg, adjusted according to response, to be given over at least 60 s, a dose of 2 mg/Kg usually produces 5-1o minutes of surgical [24] Continuous Iv Infusion: Child 1 month-17 years: starting dose 40 mcg/Kg/h., increase according to response, usual maximum dose is 100 mcg/Kg/h [26] Intranasal: Child 1 month-17 years: 0,5–10 mg/kg/dose, start with lower dose and increase according to response [28–31]
KETOROLAC	The safety and efficacy in children has not been established. The use of the drug is therefore contraindicated below 16 years. Pill and drop: used to treat short term (max 5 days) of moderate postoperative pain. Iv/im: indicated in the short term treatment (maximum two days) for moderate-severe postoperative pain. By mouth/im use for treatment of acute pain starting from 16 years of life, iv from 6 months.	By mouth and sublingual use for chidlren 4–15 years old, for a maximum period of 5 days, in patients receiving PPC without vascular access, for management of moderate/severe acute episodic nociceptive pain, which integrate other analgesia if not effective, in the course of pathology eligible to PPC or in terminall illness.	Iv: Child 6 months-15 years: initially 0,5–1 mg/Kg (max. Per dose 15 mg), than 500 mcg/Kg every 6 h (max. Per dose 15 mg) as required for maximum duration of treatment 2 days; maximum 60 mg per day [24] Im e Iv: Child 16–17 years (body-weight up to 50 Kg): initially 10 mg, than 10–30 mg every 4–6 h as required for maximum duration of treatment 2 days, maximum 60 mg per day Child 16–17 years (body-weight 50 Kg and above): initially 10 mg, than 10–30 mg every 4–6 h as required for maximum duration of treatment 2 days, maximum 90 mg per day [24] By mouth/sublingual: Child 4–15 years: 0,5 mg/Kg/ dose, maximum 3 doses/day for maximum duration of treatment 5 days [32]
LIDOCAINE	Peripheral and regional anesthesia, surgical stomatology.	1.Nebulized use for the treatment of cough refractory to other therapies, if pulmonary metastases 2. Intravenous use to treat neuropathic pain in patients in PPC not responsive to conventional therapies.	Nebulized: 5 ml of 0,2% solution every 8 h [33] Iv: 10–15 mcg/Kg/min [34]
MIDAZOLAM	Iv: conscious sedation before and during diagnostic or therapeutic procedures with or without local anaesthesia; Anesthesia: Premedication before induction of anesthesia; Sedation in ICU. Children 3 months-18 years: treatment of acute prolonged seizures Children > 1 month: treatment of status epilepticus or following crises	1. Intranasal use due to less invasiveness and high speed of administration in the absence of venous access, even in urgent cases in patients aged over 1 month in PPC. 2. Intravenous use to manage non-painful distress symptoms during end of life.	Intranasal: 0,2–0,5 mg/Kg/dose [35, 36] Iv: Child 1 month-17 years: 0,05–0,3 mg/Kg/h [26]
ONDANSETRON	Pill, syrup, vial in children ≥6 months to control chemotherapy-induced nausea and vomiting (CINV). Vial in children ≥1 month for prevention and treatment of post-operative nausea and vomiting (PONV).	Control of nausea and vomiting during opioid therapy in patients aged > 6 months in palliative care	Iv: Child 1–12 years: 5 mg/m2 (maximum single dose 8 mg) every 8–12 h Child 12–17 years: 8 mg every 8–12 h [26] By mouth Child 6 months-17 years (BSA up to 0.6 m2): 2 mg every 12 h for up to 5 days (dose can be started 12 h after iv administration); maximum 32 mg per day. Child 6 months-17 years (BSA 0.6–1.2 m2): 4 mg every 12 h for up to 5 days (dose can be started 12 h after iv administration); maximum 32 mg per day. Child 6 months-17 years (BSA 1.3 m2 and above): 8 mg every 12 h for up to 5 days (dose can be started 12 h after iv administration); maximum 32 mg per day [24]
SCOPOLAMINE	Not marketed in Italy	Treatment of hypersalivation in patients in palliative care and end of life by transdermal route.	Neonate: quarter of a patch every 72 h Child 1 month to 3 years: quarter of a patch every 72 h Child 3–10 years: half a patch every 72 h Child 11–17 years: one patch every 72 h [26]

Abbreviation: *Iv* Intravenous, *In* Intranasal, *Im* Intramuscular, *BSA* Body surface area, *ICU* Intensive Care Unit, *PPC* Pediatric Palliative Care

## Conclusions

The Agenzia Italiana del Farmaco (AIFA) and the Italian Society of Palliative Care (SICP), under the dedicated work table, drew up a document that gathers the scientific evidence available to support the off-label use of medicines more frequently used in the field of pediatric palliative care, with the goal of attesting to the off-label use of these drugs and propose their use under the law 648/96, in the absence of data from clinical trials.

## Abbreviations

AIFA: Agenzia Italiana del Farmaco
BSA: Body surface area
ICU: Intensive care unit
Im: Intramuscular
In: Intranasal
Iv: Intravenous
PPC: Pediatric palliative care
RCT: Randomized controlled trial
SICP: Società Italiana cure palliative
